# Transcriptome Analysis Provides Insights into Hepatic Responses to Trichloroisocyanuric Acid Exposure in Goldfish (*Carassius auratus*)

**DOI:** 10.3390/ani11102775

**Published:** 2021-09-23

**Authors:** Shun Zhou, Jing Dong, Yongtao Liu, Qiuhong Yang, Ning Xu, Yibin Yang, Xiaohui Ai

**Affiliations:** 1 Yangtze River Fisheries Research Institute, Chinese Academy of Fishery Sciences, Wuhan 430223, China; zhoushun@yfi.ac.cn (S.Z.); joingdong@hotmail.com (J.D.); fishliuo@hotmail.com (Y.L.); qiuhooyang@hotmail.com (Q.Y.); ningjixu@hotmail.com (N.X.); yangyooy@hotmail.com (Y.Y.); 2 Hubei Province Engineering and Technology Research Center of Aquatic Product Quality and Safety, Wuhan 430223, China

**Keywords:** histopathology, metabolism, oxidative stress, transcriptome, trichloroisocyanuric acid

## Abstract

**Simple Summary:**

Trichloroisocyanuric acid (TCCA) has been widely used in public health and aquaculture for the prevention and treatment of diseases. As a strong oxidative disinfectant, TCCA may cause adverse influences on aquatic organisms and further poses a threat to the aquatic ecosystems. Nonetheless, the toxicological influences of TCCA on aquatic animals are still scarce and the mechanisms of the toxicity at the molecular levels in goldfish (*Carassius auratus*) have not been illustrated. The current study investigated the influences of sublethal concentration of TCCA on transcriptomic responses, the molecular indices of oxidative stress, and histopathological alterations in the hepatic and gill tissues of goldfish. The results indicated that TCCA exposure induced the disturbance of energy metabolism and the detoxification process. Furthermore, TCCA exposure also induced oxidative stress in the liver and caused pathological damage in gills. These findings could be useful to help understand the toxicological influences of TCCA on goldfish.

**Abstract:**

In this study, goldfish (*Carassius auratus*) were exposed to 0 (control group) and 0.81 mg/L TCCA for four consecutive days. The liver transcriptome, the molecular indices of oxidative stress, and gills histopathology were investigated. Kyoto Encyclopedia of Genes and Genomes (KEGG) analysis indicated that energy metabolism-related pathways such as glycolysis/gluconeogenesis were significantly enriched, suggesting their perturbation in the liver of goldfish. Additionally, TCCA exposure also caused pathological damage in gills, which compromised physiological function and decreased oxygen intake capacity of gills, thus leading to the enhancement of anaerobic metabolism. This finding was confirmed by the significant upregulation of lactate dehydrogenase in the liver of goldfish. Moreover, many phase I and phase II metabolic enzymes might be activated to alleviate TCCA-induced toxicity in goldfish, and glutathione S-transferases (GSTs) and cytochrome P450s (CYPs) play a crucial role in the metabolism of TCCA in the liver of goldfish. Furthermore, the antioxidant enzyme analysis showed that TCCA exposure induced oxidative damage in the liver and partially impaired the antioxidant defense system of goldfish, evidenced by decreased superoxide dismutase (SOD) and catalase (CAT), and increased malondialdehyde (MDA) level. In summary, this study will improve our understanding of the molecular mechanisms of the TCCA-induced toxicity in goldfish.

## 1. Introduction

Chlorine-based disinfectants have been widely used in aquaculture to prevent and treat bacterial diseases due to their high efficiency, broad-spectrum, and environmental friendliness [1,2]. Trichloroisocyanuric acid (TCCA) is a common chlorinating agent with the molecular formula (C_3_Cl_3_N_3_O_3_) [3]. This agent is an unstable organic compound and reacts in water releasing the main active ingredient, hypochlorous acid. Due to its relatively small molecular weight, this compound penetrates the plasma membrane relatively easily, where it reacts with nucleotides, fatty acids, and proteins, leading to the death of cells [4]. Hypochlorous acid can also induce the inactivation of cytochromes in electron-transport systems and result in lipid peroxidation [5]. Although TCCA has potent bactericidal properties, this oxidant also exhibits varying degrees of toxicity to non-target organisms [6]. Thus, frequent and overdosage use of TCCA in aquaculture may cause adverse influences on aquatic organisms and further poses a threat to the aquatic ecosystems.

A previous study has demonstrated that high concentrations of TCCA exposure induced oxidative stress and increased the production of reactive oxygen species (ROS) in the freshwater alga *Chlorella vulgaris* [6]. Short-term exposure to a high concentration of TCCA caused DNA strand breaks and thus induced cytotoxicity and genotoxicity on the RTG-2 cell line [7]. Additionally, the main active ingredient of TCCA, hypochlorous acid and its sodium salt, have been reported to exhibit some toxicity to aquatic animals. For example, exposure to sodium hypochlorite induced the alteration in the xenobiotic metabolism and generated oxidative stress in Senegalese sole (*Solea senegalensis*) [8]. Sublethal concentration of sodium hypochlorite exposure also caused pathological alterations in gills and affected the activity of antioxidant enzymes and the lipid peroxidation levels in the common mussel (*Mytilus galloprovincialis*) [9]. However, the basic information about the physiological response of aquatic organisms after exposure to TCCA is inadequate, and the underlying molecular mechanisms of TCCA-induced toxicity against aquatic organisms have not been elucidated to date.

*Carassius auratus* has a wide distribution across Eurasia and has been an economically important fish throughout the world [10]. This species has also been becoming an important model organism in academic research due to the detailed genome information [11]. The liver is a crucial metabolic organ of fish, and plays a vital role in the detoxification and metabolism of xenobiotics. RNA-Seq is a powerful high-throughput sequencing method, which provides a better insight into the underlying molecular mechanism of TCCA-induced toxicity [12]. Therefore, in the current study, to explore the effects of TCCA exposure on goldfish, the transcriptome analysis of goldfish liver was conducted. Simultaneously, the molecular indices of oxidative stress (superoxide dismutase (SOD), catalase (CAT), glutathione S-transferase (GST), and malondialdehyde (MDA)) of goldfish liver were also investigated. Furthermore, the histological changes of goldfish gills, an organ highly exposed to xenobiotics in the aquatic environment, were also analyzed. These results would help to understand the underlying molecular mechanisms of TCCA-induced toxicity in goldfish.

## 2. Materials and Methods

### 2.1. Fish and Chemicals

A batch of juvenile goldfish were purchased from a local fish farm (Hanjin Ornamental Fish Farm, Wuhan, China) and acclimated to laboratory conditions for two weeks prior to experiments. All goldfish were maintained in 100 × 60 × 80 cm tanks containing 300-L dechlorinated water at 23 ± 1 °C under a photoperiod 10:14 h light/dark. These fish were fed twice daily with commercial pellet feed at about 2% of the total fish weight, and uneaten feed and feces were removed in 30 min. Trichloroisocyanuric acid (TCCA, 98.5% purity, J&K^®^) was obtained from Beijing Bailingwei Chemical Technology Co. Ltd. (Beijing, China).

### 2.2. Experimental Design and Sampling

According to the 48 h-LC_50_ (4.07 mg/L) of TCCA on goldfish, the exposure concentration of 0.81 mg/L TCCA (20% 48 h-LC_50_) was chosen for further study to investigate its toxicity effects on goldfish [13]. A control group without TCCA was set up under the same conditions. A total of 180 healthy goldfish with a mean bodyweight of 5.49 ± 0.73 g (mean ± SD) were randomly assigned into two groups (3 tanks per group, 30 goldfish per tank). The experiments were conducted under semi-static conditions for four consecutive days. To maintain the nominal concentrations of TCCA, the test solutions were renewed daily. The other conditions were in accordance with those during the acclimation period (water temperature, 23 ± 1 °C; pH, 6.8–7.3; dissolved oxygen, 6.1 ± 0.3 mg/L and light:dark, 10 h:14 h).

Subsequently, three individual goldfish from each group at the 1st, 2nd, and 4th day post-exposure were randomly selected, anesthetized on ice, and then liver tissues were collected (n = 18; 3 samples per sampling time point per group). Each liver tissue was divided into two portions: one for the determination of enzyme activity and one for RNA extraction. These samples were frozen in liquid nitrogen and then stored at −80 °C until used. The samples collected on the fourth day after exposure were used for the transcriptome sequencing. In addition, gill tissues from three individuals of each group were collected at the 1st, 2nd, and 4th day post-exposure and fixed in a 4% paraformaldehyde solution for histopathological analysis.

### 2.3. RNA Extraction and Transcriptome Sequencing

Four liver samples from the control and treatment groups collected on the fourth day after exposure were selected for the transcriptome sequencing. Total RNA was extracted using TRlzol Reagent (Life Technologies, Carlsbad, CA, USA) and the concentration and integrity of total RNA were evaluated using NanoDrop2000 spectrophotometer (Thermo Fisher Scientific, Wilmington, DE, USA) and Agilent Bioanalyzer 2100 (Agilent Technologies, Santa Clara, CA, USA). A total amount of 1 μg RNA per sample was used for the RNA-seq libraries construction with NEBNext UltraTM RNA Library Prep Kit for Illumina (NEB, Ipswich, MA, USA). The constructed libraries were sequenced on an Illumina NovaSeq 6000 platform with paired-end reads.

### 2.4. Bioinformatics Analysis

The adaptor sequences and low-quality sequence reads were removed from raw reads to obtain clean reads. These clean reads were then mapped to the reference genome of goldfish (https://www.ncbi.nlm.nih.gov/assembly/GCF_003368295.1/) (accessed on 7 April 2021) using the Hisat2 tools soft. Gene expression levels were estimated by fragments per kilobase of transcript per million fragments mapped [14]. Differential expression analysis was performed using the DESeq2 with a corrected *p*-value < 0.05 as a threshold for significance. The *p* values were adjusted using Benjamini and Hochberg’s approach for controlling the false discovery rate (FDR), and only the genes with |log2 (fold change)| > 2 and FDR < 0.01 were selected for further analysis. Gene Ontology (GO) enrichment analysis of the differentially expressed genes (DEGs) was implemented by the GOseq R packages and Kyoto Encyclopedia of Genes and Genomes (KEGG) pathway enrichment analysis was conducted using KOBAS software [15,16]. The pathways or GO terms with an adjusted *p*-value < 0.05 were considered to be significantly enriched pathways or GO terms.

### 2.5. Quantitative Real-Time Polymerase Chain Reaction (qPCR)

To validate the reliability of the RNA-Seq results, ten DEGs were selected for qPCR confirmation based on different expression patterns with functional enrichment and pathway results. The primers (Table S1) for these DEGs were designed using the Primer3 software (http://primer3.ut.ee/; accessed on 18 May 2021). The qPCR assays were carried out using a QuantStudio^TM^ 3 real-time PCR System. The thermal cycle was as follows: 30 s at 95 °C, followed by 40 cycles of denaturing at 95 °C for 10 s, and annealing at 60 °C for 30 s. The β-actin gene was used as an internal reference gene, and the relative expression level of these selected DEGs was quantified using a comparative CT method (2^−ΔΔCt^ method) [17].

### 2.6. Determination of Antioxidant Enzyme Activities

The liver tissue (n = 18; 3 samples per sampling time point per group) was homogenized (1:10, *w/v*) in cold physiological saline solution (0.9% NaCl) by using glass tissue homogenizers under ice-bath cooling. The homogenate was centrifuged at 4000× *g* for 15 min at 4 °C, then the supernatant was collected for biochemical analysis. The enzyme activities (SOD, CAT, and GST), MDA level and protein content in the supernatants were measured by using the commercial kits (Nanjing Jiancheng Bioengineering Institute, Nanjing, China). SOD activity was measured by the WST-1 (water-soluble tetrazolium-1) method at 450 nm [18]. CAT activity was determined by the degradation of hydrogen peroxide at 405 nm [19]. GST activities were measured at 412 nm according to the method described by Mannervik and Guthenberg [20]. MDA content was estimated based on the reaction of the generated substrate and the thiobarbituric acid at 532 nm [21]. The protein concentration was quantified according to the method of Bradford using bovine serum albumin solution as standard [22]. All data were assessed for normality and homogeneity of variance prior to further analysis and data transformations were made as necessary to fulfill the requirements of statistical analysis of variance. The statistical differences between groups were calculated using independent *t*-tests in the SPSS 20.0 software (IBM, New York, NY, USA), and the threshold for statistical significance was set at *p* < 0.05.

### 2.7. Histopathological Analysis

All gill tissues (n = 18; 3 samples per sampling time point per group) were fixed in a 4% paraformaldehyde solution for 48 h. These tissues were embedded in paraffin wax and processed by standard paraffin wax techniques. Subsequently, the sections were cut at 5 µm thickness using a rotatory microtome and then stained with hematoxylin and eosin according to a standard protocol [23]. The sections were observed under the imaging microscope and photographed using a Primo Star microscope (Carl Zeiss Microscopy GmbH, Jena, Germany).

## 3. Results

### 3.1. Transcriptome Sequence and Reads Mapping

The cDNA libraries were constructed and sequenced from goldfish livers and the characteristics of these libraries are summarized in Table S2. After quality control of sequencing data, a total of 20,774,117 and 26,822,598 clean reads in the liver were obtained from the control group, respectively, compared with 25,491,795 and 21,099,759 clean reads from the TCCA-treated group. The Q30 values of each sequencing library were higher than 95.01%, indicating good sequencing quality. These high-quality clean reads were then mapped into the reference genome with genome mapping rates over 83.62% in different libraries. Additionally, the Pearson correlation between the samples was up to 0.83 (Figure S1). During the experiment, no deaths were observed in the control and treatment groups.

### 3.2. Differential Expression Analysis and Functional Annotation

**Figure 1:**
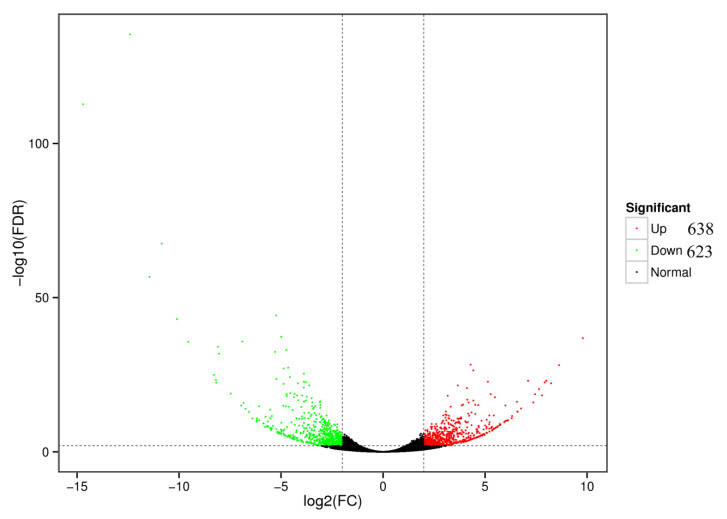
Volcano plot of distribution trends for differentially expressed genes between control groups and TCCA treatment groups. Each dot represents one gene. Red dots represent upregulated genes and green dots represent downregulated genes. Black dots represent genes with no differential expression.

According to the statistical analysis of unigenes with DESeq2, the DEGs between control group and TCCA treatment group were identified. A total of 1261 genes were differentially expressed, including 638 upregulated and 623 downregulated genes (Figure 1).

### 3.3. GO and KEGG Pathway Enrichment Analysis

**Figure 2:**
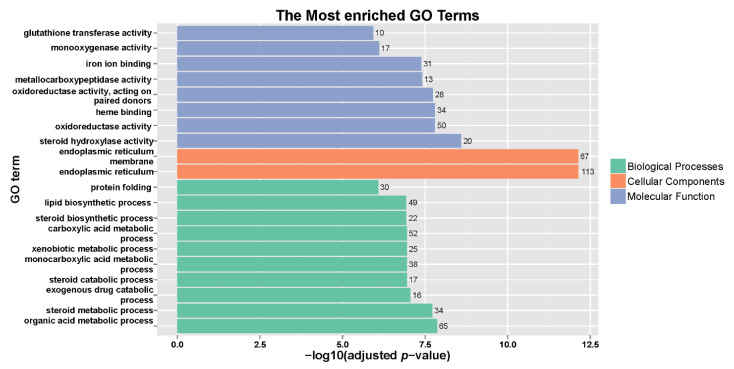
Gene ontology annotation of the differentially expressed genes in the three main GO categories: biological process, cellular component, and molecular function. The x-axis means adjusted *p*-value, the y-axis shows the top 20 most represented GO terms, and the values on the right of the columns represent the number of differentially expressed genes.

To characterize the biological function of DEGs, GO classification and KEGG pathway enrichment analysis were conducted. The GO analysis revealed that 1057 DEGs were divided into three categories in the goldfish: biological processes, cellular components, and molecular functions (Figure 2 and Supplementary Material S2). The subclasses cellular process, cell, and binding were the most significantly regulated in these three categories after TCCA exposure. The most enriched GO terms in the biological processes were organic acid metabolic process (GO:0006082), steroid metabolic process (GO:0008202), and exogenous drug catabolic process (GO:0042738). In the cellular components, the terms of endoplasmic reticulum (GO:0005783), endoplasmic reticulum membrane (GO:0005789), and endoplasmic reticulum part (GO:0044432) were also significantly enriched. In the molecular functions, the most enriched GO terms were steroid hydroxylase activity (GO:0008395), oxidoreductase activity (GO:0016491), and heme-binding (GO:0020037).

**Figure 3:**
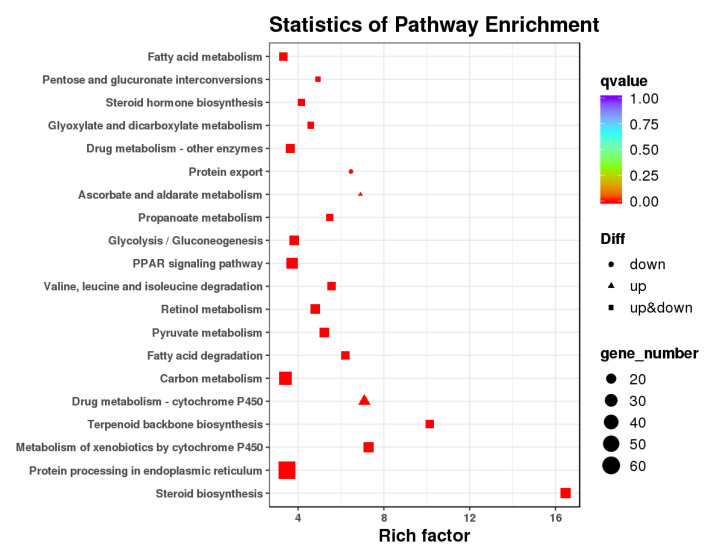
Top 20 statistics of pathway enrichment based on the differentially expressed genes in goldfish after exposure to trichloroisocyanuric acid. Rich Factor is the ratio of differentially expressed gene numbers noted in this pathway term to all gene numbers noted in this pathway term; the “up” indicates that only upregulated genes are enriched in this pathway, “down” indicates that only downregulated genes are enriched in this pathway, and “up and down” indicates that both upregulated and downregulated genes are enriched in this pathway. All top pathways had a q-value less than 0.0001.

According to the KEGG pathway analysis, 612 DEGs were mapped to 177 pathways in the KEGG database, and 40 pathways were significantly enriched (Supplementary Material S3). The twenty highly enriched categories of KEGG are presented in Figure 3, and these pathways were mainly involved in metabolism, genetic information processing, and organismal systems (Supplementary Material S3). Among them, the number of DEGs associated with metabolism was the largest, including carbohydrate metabolism, lipid metabolism, and xenobiotics biodegradation and metabolism. Specifically, a total of 95 DEGs were enriched into carbohydrate metabolism processes, including glycolysis/gluconeogenesis, pyruvate metabolism, propanoate metabolism, ascorbate and aldarate metabolism, glyoxylate and dicarboxylate metabolism, and pentose and glucuronate interconversions. A total of 73 DEGs were enriched into lipid metabolism processes, including steroid biosynthesis, fatty acid degradation, steroid hormone biosynthesis, and fatty acid metabolism. These pathways are involved in energy metabolism, and some representative DEGs of these pathways are presented in Table S3. Additionally, 63 DEGs were enriched into xenobiotics biodegradation and metabolism processes, including metabolism of xenobiotics by cytochrome P450, drug metabolism–cytochrome P450, and drug metabolism–other enzymes (Table S4). Furthermore, some other detoxification-related DEGs were also detected in goldfish after exposure to TCCA (Table S4).

### 3.4. Validation of RNA-Seq Results with qPCR

**Figure 4:**
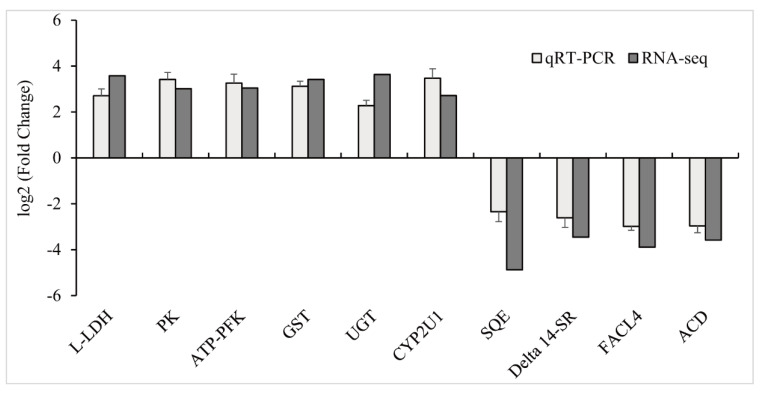
Differentially expressed genes in goldfish liver confirmed by qPCR. The X–axis displays 10 DEGs and the Y–axis represents relative fold change. The data are expressed as the means ± SD (n = 3).

To validate the results of RNA-seq, ten DEGs were selected for qPCR analysis. As shown in Figure 4, the expression value of these genes was in good agreement with the sequencing results. The results demonstrated that RNA-Seq results were reliable.

### 3.5. Analysis of Antioxidant Enzyme Activities

**Figure 5:**
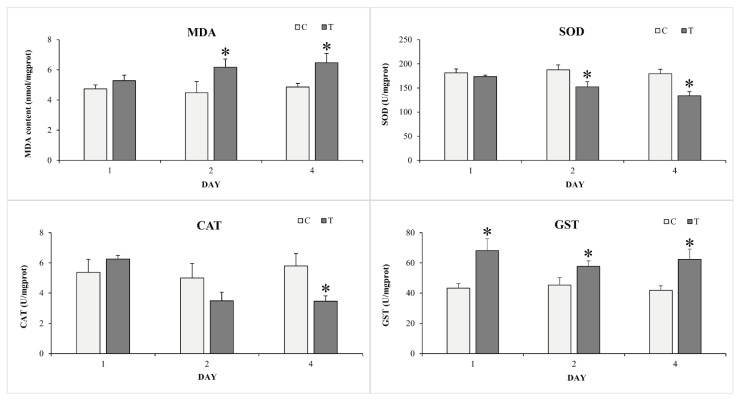
The MDA content and antioxidant enzyme activities (SOD, CAT, and GST) in the liver of goldfish during four days of trichloroisocyanuric acid treatment. Each bar represents mean ± SD (n = 3) and the asterisk indicates significant differences (*p* < 0.05).

The antioxidant enzyme activities and MDA content in the liver of goldfish after exposure to TCCA are summarized in Figure 5. No significant difference was found in the enzyme activities and MDA content at different sampling points in the control group (1st, 2nd, and 4th day post-exposure). Compared with the control group, the SOD activity did not significantly change on the first day, but was significantly reduced in the second and fourth days in the liver of goldfish (*p* < 0.05). The CAT activity was increased on the first day but significantly decreased on the fourth day (*p* < 0.05). On the contrary, the GST activity was significantly increased during all exposure periods (*p* < 0.05). The MDA content was significantly increased in the second and fourth days after exposure to TCCA (*p* < 0.05).

### 3.6. Histopathological Analysis after Exposures to TCCA

**Figure 6:**
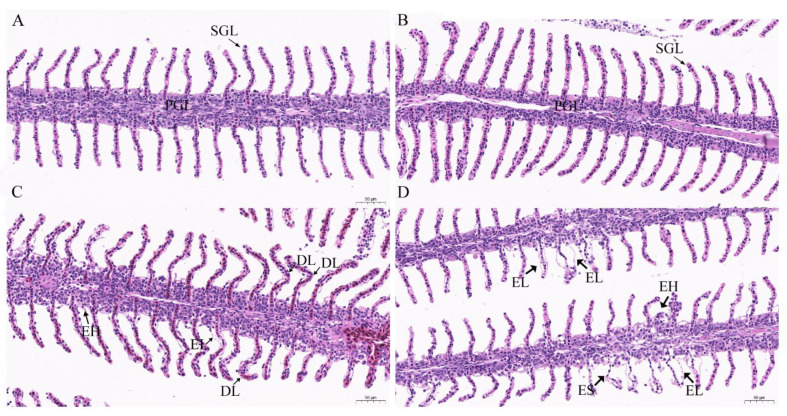
Histopathological analysis in the gills of goldfish (*Carassius auratus*) after exposure to 0.81 mg/L trichloroisocyanuric acid. (**A**) goldfish gills of the control group (0 mg/L TCCA), primary gill lamellae (PGL) and secondary gill lamellae (SGL); (**B**) goldfish gills exposed to 0.81 mg/L TCCA for one day, primary gill lamellae (PGL) and secondary gill lamellae (SGL); (**C**) goldfish gills exposed to 0.81 mg/L TCCA for two days, epithelial hyperplasia (EH), epithelial lifting (EL), and deformed lamellae (DL); (**D**) goldfish gills exposed to 0.81 mg/L TCCA for four days, epithelial hyperplasia (EH), epithelial lifting (EL), and epithelial shedding (ES).

Histological analysis of goldfish gills exposed to TCCA was displayed in Figure 6. In the control group, a normal morphological structure with primary gill lamellae and secondary gill lamellae was observed (Figure 6A). A similar result was observed for goldfish gills exposed to TCCA for one day (Figure 6B). Epithelial hyperplasia, epithelial lifting, and deformed lamellae appeared in the goldfish gills exposed to TCCA for two days (Figure 6C). In addition, epithelial hyperplasia, epithelial lifting, and epithelial shedding were observed in goldfish gills exposed to TCCA for four days (Figure 6D).

## 4. Discussion

Trichloroisocyanuric acid, as a strong oxidative disinfectant, has been widely used in public health and aquaculture for the prevention and treatment of diseases [6,24]. However, comprehensive studies of the effects of TCCA on aquatic animals are still scarce. In the present study, the alterations of the liver transcriptomic profile, the molecular indices of oxidative stress, and gills histopathology of goldfish were investigated after acute exposure to TCCA, aiming to provide a comprehensive understanding of the underlying molecular mechanisms of TCCA-induced toxicity in goldfish.

### 4.1. Effects of TCCA Exposure on Energy Metabolism

In this study, the results of transcriptome analysis indicated that energy metabolism-related pathways such as glycolysis/gluconeogenesis, pyruvate metabolism, and fatty acid metabolism, were significantly enriched, suggesting their perturbation in the liver of goldfish treated with TCCA. Among these pathways, glycolysis/gluconeogenesis is one of the most significantly affected pathways with 22 DEGs involved. Most of the DEGs (Table S3) enriched in this pathway were significantly upregulated, including 6-phosphofructokinase and pyruvate kinase, which are the crucial rate-limiting enzymes regulating glycolysis [25]. The up-regulation of these genes indicated glycolysis process was initiated to meet the energy demand in the liver of goldfish after exposure to TCCA. Lactate dehydrogenase, an important indicator of anaerobic metabolism, can catalyze the formation of lactic acid from pyruvate during anaerobic metabolism [26]. In this trial, the expression level of lactate dehydrogenase was significantly upregulated, suggesting the enhancement of anaerobic metabolism. In the anaerobic metabolism, glucose is converted to pyruvate and lactic acid, which leads to the accumulation of lactic acid [27]. The finding was supported by a previous study, which has demonstrated that the lactate levels were significantly elevated in Senegalese sole exposed to chlorine-based disinfectant-sodium hypochlorite [8]. These results indicated that exposure to TCCA was able to induce a hypoxic state on goldfish, then cause a series of anaerobic metabolism. The possible explanation for such results might be related to the histopathological damage in the gills induced by TCCA exposure. The damage could compromise physiological function and decrease oxygen intake capacity of gills, thus leading to the enhancement of anaerobic metabolism.

### 4.2. Effects of TCCA Exposure on Detoxification Responses

The liver is the major organ for the metabolism and detoxification of xenobiotics in teleost fishes [28]. Previous studies have indicated that detoxification-related pathways and genes were differentially regulated in the liver of aquatic animals after exposure to xenobiotics, such as methylene blue and formalin [29,30]. In the present study, several detoxification pathways, such as drug metabolism–cytochrome P450, drug metabolism–other enzymes, and metabolism of xenobiotics by cytochrome P450, were significantly regulated in goldfish after exposure to TCCA. Among these pathways, most of the detoxification-related genes, such as GSTs, UDP-glucuronosyltransferase, epoxide hydrolase, carbonyl reductase, and flavin-containing monooxygenase, were significantly upregulated (Table S4). GSTs are significant enzymes for the metabolism of xenobiotics in phase II, which can catalyze the conjugation of glutathione to electrophilic centers of xenobiotic substrates for detoxification [31]. Epoxide hydrolase, carbonyl reductase, and flavin-containing monooxygenase are important phase I detoxification enzymes, which might convert xenobiotics into less toxic and non-toxic compounds [32,33]. The significant upregulation of these genes suggested the detoxification process mediated by these genes could be initiated in goldfish in response to TCCA. Additionally, significant upregulations in several cytochrome P450 (CYPs) were also observed in goldfish after exposure to TCCA. Similarly, the activities of several CYP-metabolizing enzymes in the livers of common carp (*Cyprinus carpio*) were significantly increased after exposure to sodium hypochlorite and chlorine dioxide [34]. CYPs are a superfamily of heme-containing monooxygenases that are involved in phase I detoxification of many endogenous and exogenous substances [35]. These results indicated many phase I and phase II metabolic enzymes might be activated to alleviate TCCA-induced toxicity in goldfish, and GSTs and CYPs play a crucial role in the metabolism of TCCA in the liver of goldfish.

### 4.3. TCCA Exposure Induced Oxidative Damage in Liver

ROS are indispensable for normal cellular function and play an important role in defense against a wide range of pathogens [36]. However, excessive ROS could induce oxidative damage to cells and tissues, resulting in an imbalance of normal physiologic function [37]. MDA, an end product of lipid peroxidation (LPO), has been used as a key biomarker to reflect oxidative stress and tissues damage [38]. In the present study, MDA levels increased significantly in the second and fourth days after exposure to TCCA, which demonstrated oxidative stress effects of TCCA exposure in goldfish. To clear excessive ROS, organisms have developed complicated antioxidant defense systems, such as the antioxidant enzymes SOD and CAT. SOD catalyzes the conversion of superoxide radical (O_2_^−^) to hydrogen peroxide (H_2_O_2_) and molecular oxygen (O_2_), while CAT decomposes H_2_O_2_ into O_2_ and molecules of water (H_2_O) to eliminates free radicals [39]. Previous studies have indicated that exposure to chlorine-based disinfectants suppressed the activities of antioxidant enzymes in fish [2,8]. The activity of SOD in rainbow trout (*Oncorhynchus mykiss*) was significantly decreased after exposure to 5 mg/L chloride dioxide [2]. Lopez-Galindo et al. (2010) have reported that long-term exposure to sodium hypochlorite caused a significant decrease in CAT activity in Senegalese sole [8]. Similarly, in this trial, significant decreases in the levels of SOD and CAT were observed in the liver of goldfish after exposure to TCCA for four days. These results indicated exposure to TCCA might inhibit the antioxidant defense of goldfish. Nevertheless, the evident increase in CAT activity following a 1-day exposure to TCCA might be due to increased endogenous H_2_O_2_, which might also be an adaptive response to oxidative stress. In addition, the activity of GST was significantly increased during all exposure periods. The finding was supported by the transcriptome result that the expression levels of several GSTs were significantly upregulated in the liver after exposure to TCCA. Similar results have been reported for other chlorine-based disinfectants, which induced significant increases in GST activity in common carp [40]. These results indicated the GST enzyme system was activated to protect the organism from damage caused by oxidative stress. Overall, TCCA exposure induced excessive ROS production and partially impaired the antioxidant defense system of goldfish.

### 4.4. TCCA Exposure Induced Histopathological Damage in Gill

In addition to regulating metabolism-related genes expressions and antioxidant defense systems in fish, TCCA also induced histological alterations in gills, which are continuously in direct contact with xenobiotic contaminants in aquatic environments. Various histological alterations, including epithelial hyperplasia, epithelial lifting, epithelial shedding, and deformed lamellae in the gills of goldfish were observed after exposure to TCCA. Similar findings were reported in Senegalese sole and *M. galloprovincialis* after exposure to sublethal concentrations of chlorine-based disinfectant-sodium hypochlorite [8,9]. Additionally, Yonkos et al. (2000) showed that exposure to another chlorine-based disinfectant—chlorine dioxide–resulted in epithelial lifting, hypertrophy, hyperplasia, lamellar fusion, and necrosis in the gills of fathead minnows (*Pimephales promelas*) [41]. Therefore, exposure to chlorine-based disinfectants such as TCCA can induce histopathological damage in the gills of aquatic animals. Gills serve as the primordial respiratory organs and the histopathological damage induced by TCCA exposure might interfere with the normal physiologic functions.

## 5. Conclusions

In summary, this study indicated that exposure to TCCA has deleterious effects on goldfish. Transcriptome analysis indicated that several energy metabolism-related pathways and DEGs were significantly enriched, suggesting a sublethal concentration of TCCA induced a perturbation of energy metabolism in the liver of goldfish. Additionally, TCCA exposure induced excessive ROS production and partially impaired the antioxidant defense system of goldfish. Moreover, TCCA exposure also caused pathological damage in gills, which might interfere with the normal physiologic functions. However, in the current study, the limited biological replications per treatment resulted in low detection power for the differential expression. Despite the limitation, this study could also improve our understanding of the molecular mechanisms of the TCCA-induced toxicity in goldfish.

## Data Availability

The datasets used and analyzed during the current study are available from the corresponding author on reasonable request.

## Supplementary Materials

The following are available online at https://www.mdpi.com/article/10.3390/ani11102775/s1, Table S1: The primers used in the quantitative PCR analysis, Figure S1: Heatmap of Pearson correlation between samples. Both X and Y-axis represent each sample. Coloring indicates Pearson correlation. C1 and C2 represent the control samples, T1 and T2 represent the samples treated with 0.81 mg/L trichloroisocyanuric acid, Table S2: Summary statistics of the transcriptome sequences. C: 0 mg/L trichloroisocyanuric acid; T: 0.81 mg/L trichloroisocyanuric acid, Table S3: Representative energy metabolism-related pathways and differentially expressed genes in goldfish after treated with trichloroisocyanuric acid, Table S4: Representative detoxification-related pathways and differentially expressed genes in goldfish after treated with trichloroisocyanuric acid, Supplementary Material S2: the results of GO classification analysis, Supplementary Material S3: the results of KEGG pathway enrichment analysis, Supplementary Material S4: all DEGs and the top 50 DEGs between control and TCCA-treated groups.
