# Supplementary material for: Transcriptome Analysis Provides Insights into Hepatic Responses to Trichloroisocyanuric Acid Exposure in Goldfish (Carassius auratus)
Source: Animals (Basel). 2021 Sep 23;11(10):2775. doi: 10.3390/ani11102775 (PMC8532840; doi:10.3390/ani11102775)
Supplement: Supplementary file 1 [file animals-11-02775-s001.zip › Supplementary material S1.pdf]

Supplementary material S1

Transcriptome analysis provides insights into hepatic responses to trichloroisocyanuric acid exposure in goldfish (*Carassius auratus*)

Shun Zhou[1,2], Jing Dong[1,2], Yongtao Liu[1,2], Qiuhong Yang[1,2], Ning Xu[1,2], Yibin Yang[1,2], Xiaohui Ai[1,2*]

[1] Yangtze River Fisheries Research Institute, Chinese Academy of Fishery Sciences, Wuhan, 430223, China

[2] Hu Bei Province Engineering and Technology Research Center of Aquatic Product Quality and Safety, Wuhan, 430223, China

[*]Author for correspondence: Xiaohui Ai,

Yangtze River Fisheries Research Institute, Chinese Academy of Fishery Sciences,

No. 8 Wuda Park Road 1, Wuhan 430223, Hubei Province, China

E-mail address: aixh@yfi.ac.cn

Table S1. The primers used in the quantitative PCR analysis.

Name	Forward primers (5’-3’)	Reverse primers (5’-3’)
L-LDH	GGTGAAGGTGAGCGGAGTTT	GCCCTGCAGAAGACACCATA
PK	GGTGCATCATGGGTGATTCTG	CGCTGCAACACACTCAACATC
ATP-PFK	ACGCCGTCTAGTGATCATGT	ATTCCTATCGAACGGAGTGGG
GST	TAAAGGCTCGTACCTCGCTG	GCTGACCTGAATGTGTTATGAGG
UGT	ATCACTCATGGTGGAACGCA	CATCAAACTCTCACCTGCCGT
CYP2U1	GCCAGTGGTGGAAATCCTTGA	TGGCATCGTGCTGTGTGATA
SQE	GACAGTACGGCACCTCAACA	TGTCACTTACTGTCGGTCGC
Delta 14-SR	GTTGGTATCAGGGTGGTGGG	ACATGAATGAATAGTGCTACTGTGA
FACL4	GATCTGCAACCACCCGGTTA	TCACCAACAAACCTTGCTGC
ACD	GTATTTCGTCCCTGCCCTCC	AGAACATCAGACGTACCGATAGC
β-actin	GATGATGAAATTGCCGCACTG	ACCGACCATGACGCCCTGATGT

Abbreviations: L-LDH, L-lactate dehydrogenase; PK, pyruvate kinase PKM; ATP-PFK, ATP-dependent 6-phosphofructokinase, muscle type-like; GST, glutathione S-transferase; UGT UDP-glucuronosyltransferase 1-5-like; CYP2U1, cytochrome P450 2U1; SQE, squalene monooxygenase-like; Delta 14-SR, delta(14)-sterol reductase-like; FACL4, long-chain-fatty-acid--CoA ligase 4-like isoform X1; ACD, acyl-CoA desaturase-like.

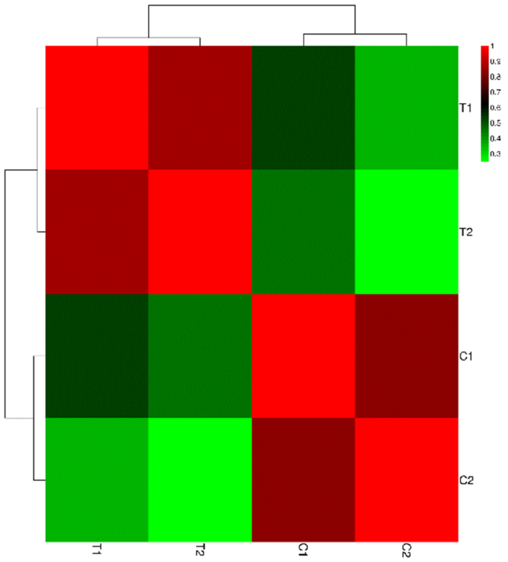

Figure S1. Heatmap of Pearson correlation between samples. Both X and Y-axis represent each sample. Coloring indicates Pearson correlation. C1 and C2 represent the control samples, T1 and T2 represent the samples treated with 0.81 mg/L trichloroisocyanuric acid.

Table S2. Summary statistics of the transcriptome sequences. C: 0 mg/L trichloroisocyanuric acid; T: 0.81 mg/L trichloroisocyanuric acid.

Sample	Clean reads	Clean bases	GC content (%)	Q30 (%)	Total Mapped reads (ratio)	Multiple mapped reads (ratio)	Uniquely mapped reads (ratio)
C1	20,774,117	6,222,361,568	45.64%	95.01%	36,279,169 (87.32%)	32,522,911 (78.28%)	3,756,258 (9.04%)
C2	26,822,598	8,027,048,978	45.75%	95.95%	44,855,638 (83.62%)	39,772,028 (74.14%)	5,083,610 (9.48%)
T1	25,491,795	7,632,550,880	46.27%	95.93%	43,445,910 (85.22%)	39,885,362 (78.23%)	3,560,548 (6.98%)
T2	21,099,759	6,313,811,726	46.70%	95.85%	37,238,257 (88.24%)	34,152,239 (80.93%)	3,086,018 (7.31%)

Table S3. Representative energy metabolism-related pathways and differentially expressed genes in goldfish after treatment with trichloroisocyanuric acid.

Gene ID	Gene description	Change	log2FoldChange	FDR
**Glycolysis/Gluconeogenesis**				
gene-aldh2	aldehyde dehydrogenase, mitochondrial-like	up	2.04	8.83E-05
gene-LOC113048028	L-lactate dehydrogenase	up	3.58	3.09E-08
gene-LOC113065773	enolase-like	up	2.23	2.51E-05
gene-LOC113048782	pyruvate dehydrogenase E1 component subunit alpha, mitochondrial-like isoform X1	down	-6.90	2.02E-36
gene-LOC113048182	L-lactate dehydrogenase	up	2.85	0.0027965
gene-LOC113057076	alcohol dehydrogenase class-3	up	4.12	1.13E-08
gene-LOC113072161	alcohol dehydrogenase class-3-like isoform X4	up	4.58	4.08E-08
gene-LOC113107227	4-trimethylaminobutyraldehyde dehydrogenase A-like	up	4.03	1.28E-10
Carassius_auratus_newGene_9683	acetyl-coenzyme A synthetase 2-like, mitochondrial isoform X1	down	-4.04	1.06E-06
gene-LOC113059429	pyruvate kinase PKM	up	3.01	9.18E-05
gene-LOC113113586	aldolase class-1 protein	up	2.53	0.0016387
gene-LOC113081964	phosphoenolpyruvate carboxykinase-like: cytosolic [GTP] isoform X1	down	-4.10	1.79E-11
gene-LOC113113588	aldolase class-1 protein	up	3.02	3.67E-08
gene-LOC113111269	ATP-dependent 6-phosphofructokinase, muscle type-like	up	3.04	3.30E-08
gene-LOC113117432	acetyl-coenzyme A synthetase 2-like, mitochondrial isoform X4	down	-2.80	6.15E-08
gene-LOC113070452	fructose-1,6-bisphosphatase 1	up	2.20	2.04E-05
gene-LOC113112835	alcohol dehydrogenase class-3	up	3.35	5.34E-11
**Propanoate metabolism**				
gene-ehhadh	peroxisomal bifunctional enzyme-like	up	2.97	2.40E-06
gene-LOC113048915	acetyl-CoA carboxylase-like isoform X2	down	-2.06	0.0040882
gene-LOC113060523	methylmalonate-semialdehyde dehydrogenase [acylating], mitochondrial-like	up	2.08	1.04E-07
gene-LOC113093300	malonyl-CoA decarboxylase, mitochondrial-like	up	3.48	0.0014967
**Ascorbate and aldarate metabolism**				
gene-LOC113050879	regucalcin-like	up	5.20	1.20E-09
gene-LOC113092184	regucalcin-like	up	2.32	0.0011164
**Glyoxylate and dicarboxylate metabolism**				
gene-LOC113065564	citrate synthase, mitochondrial-like	down	-2.09	2.55E-05
gene-LOC113050664	glutamine synthetase-like isoform X1	down	-2.26	1.02E-07
gene-LOC113056249	glycerol-3-phosphate phosphatase-like	down	-3.07	1.15E-07
**Pentose and glucuronate interconversions**				
Carassius_auratus_newGene_5907	UDP-glucuronosyltransferase 1-1-like	up	2.16	0.0064339

gene-LOC113076602	glucose-fructose oxidoreductase domain-containing protein 1-like isoform X1	down	-2.03	0.0028573
gene-LOC113063794	oxidoreductase, aldo/keto reductase family member protein	up	2.37	0.0004931
gene-LOC113061555	aldose reductase-like	up	2.37	0.0067061
gene-LOC113117483	UTP--glucose-1-phosphate uridylyltransferase-like isoform X1	down	-2.10	3.66E-05
gene-LOC113118076	trans-1,2-dihydrobenzene-1,2-diol dehydrogenase-like	up	2.47	5.28E-07
gene-LOC113118075	trans-1,2-dihydrobenzene-1,2-diol dehydrogenase-like	up	2.20	1.47E-06
gene-sord	sorbitol dehydrogenase-like	up	5.14	1.90E-23
gene-LOC113055297	UDP-glucuronosyltransferase 1-5-like	up	3.63	1.23E-06
**pyruvate metabolism**				
gene-LOC113069931	4-trimethylaminobutyraldehyde dehydrogenase-like	up	2.80	1.55E-08
gene-LOC113080845	glyoxylate reductase/hydroxypyruvate reductase-like	down	-2.67	2.40E-06
**Steroid biosynthesis**				
gene-LOC113038855	neuroligin-4, X-linked	up	3.41	1.06E-06
gene-LOC113079864	delta(24)-sterol reductase-like	down	-4.65	5.31E-28
gene-LOC113062498	lanosterol 14-alpha demethylase	down	-3.97	5.31E-19
gene-LOC113120553	squalene synthase-like	down	-3.32	1.50E-05
gene-LOC113113575	sterol-4-alpha-carboxylate 3-dehydrogenase, decarboxylating-like	down	-3.88	2.04E-08
gene-LOC113121083	delta(24)-sterol reductase-like	down	-2.86	2.42E-07
gene-lss	lanosterol synthase-like	down	-4.33	1.71E-19
gene-dhcr7	7-dehydrocholesterol reductase-like isoform X1	down	-2.96	1.27E-09
gene-LOC113059233	squalene monooxygenase-like	down	-4.87	8.48E-23
gene-LOC113115850	squalene monooxygenase-like	down	-2.54	0.0004226
gene-LOC113068573	squalene synthase-like	down	-2.23	0.0004751
gene-LOC113073347	squalene synthase-like	down	-2.76	0.0003244
gene-LOC113063930	neuroligin-4, X-linked	up	3.13	2.13E-06
gene-LOC113076135	delta(14)-sterol reductase-like	down	-2.16	0.000262
gene-LOC113054335	delta(14)-sterol reductase-like	down	-3.45	4.98E-06
gene-LOC113078828	lathosterol oxidase	down	-2.49	3.25E-05
gene-ebp	3-beta-hydroxysteroid-Delta(8),Delta(7)-isomerase-like	down	-4.57	5.46E-25
gene-LOC113113351	sterol-4-alpha-carboxylate 3-dehydrogenase, decarboxylating-like	down	-3.08	5.59E-06
gene-LOC113062450	lanosterol 14-alpha demethylase	down	-4.00	2.44E-15
**Fatty acid degradation**				
gene-LOC113073480	long-chain-fatty-acid--CoA ligase 4-like isoform X1	down	-3.89	1.29E-21
gene-LOC113043584	carnitine O-palmitoyltransferase 1, liver isoform-like isoform X2	up	2.07	0.0083275
gene-LOC113117377	medium-chain specific acyl-CoA dehydrogenase, mitochondrial-like	up	2.86	8.60E-07

gene-LOC113110014	acetyl-CoA acetyltransferase, mitochondrial-like	up	3.66	2.66E-22
gene-LOC113062791	acetyl-CoA acetyltransferase, cytosolic-like	down	-3.70	2.26E-07
gene-LOC113049649	long-chain-fatty-acid--CoA ligase ACSBG2-like isoform X3	down	-3.32	0.0044905
gene-LOC113120792	acetyl-CoA acetyltransferase, cytosolic-like	down	-3.80	2.12E-17
gene-LOC113067102	carnitine O-palmitoyltransferase 1, liver isoform-like isoform X2	up	3.21	0.000107
**Steroid hormone biosynthesis**				
Carassius_auratus_newGene_8844	cytochrome P450 3A24-like	up	2.40	9.35E-09
gene-LOC113107162	catechol O-methyltransferase-like	up	2.92	0.0069445
gene-LOC113083757	cytochrome P450 3A24-like	up	2.20	0.0004411
gene-comtd1	catechol O-methyltransferase domain-containing protein 1-like	up	2.07	0.0022736
gene-sts	steryl-sulfatase-like	up	4.29	5.66E-29
gene-LOC113100639	cytochrome P450 3A24-like	up	2.24	7.13E-05
gene-LOC113038900	cytochrome P450 3A24-like	up	2.67	6.98E-08
**Fatty acid metabolism**				
gene-LOC113111436	acyl-CoA desaturase-like	down	-3.58	6.56E-06
gene-LOC113077428	estradiol 17-beta-dehydrogenase 12-B-like	up	2.76	0.004865
gene-LOC113111444	elongation of very long chain fatty acids protein 6-like	up	2.13	0.0001103
gene-LOC113069873	elongation of very long chain fatty acids protein 4-like	up	2.46	0.0026433
gene-LOC113106903	HSD17B4	up	2.32	1.02E-05

Table S4. Representative detoxification-related pathways and differentially expressed genes in goldfish after treatment with trichloroisocyanuric acid.

Gene ID	Gene description	Change	log2FoldChange	FDR
**metabolism of xenobiotics by cytochrome P450**				
gene-LOC113076602	glucose-fructose oxidoreductase domain-containing protein 1-like isoform X1	down	-2.03	0.00286
Carassius_auratus_newGene_7011	epoxide hydrolase 1-like	up	2.53	0.00087
gene-LOC113119694	glutathione S-transferase	up	3.42	0.0036
gene-LOC113113088	glutathione S-transferase A4-like	up	3.58	1.57E-08
gene-LOC113098224	carbonyl reductase [NADPH] 1-like	up	4.10	0.00031
gene-LOC113057076	alcohol dehydrogenase class-3	up	4.12	1.13E-08
gene-LOC113072161	alcohol dehydrogenase class-3-like isoform X4	up	4.58	4.08E-08
gene-LOC113056744	epoxide hydrolase 1-like	up	2.37	0.00641
gene-LOC113093931	glutathione S-transferase	up	3.24	1.99E-05
gene-LOC113056663	microsomal glutathione S-transferase 3-like isoform X1	up	2.97	2.68E-06
gene-LOC113092965	microsomal glutathione S-transferase 3-like	up	3.21	1.22E-11
gene-LOC113088650	glutathione S-transferase	up	3.06	8.22E-12
gene-LOC113119695	glutathione S-transferase A-like	up	4.13	1.06E-17
gene-LOC113119696	glutathione S-transferase	up	4.12	2.29E-21
gene-LOC113118076	trans-1,2-dihydrobenzene-1,2-diol dehydrogenase-like	up	2.47	5.28E-07
gene-LOC113118075	trans-1,2-dihydrobenzene-1,2-diol dehydrogenase-like	up	2.20	1.47E-06
gene-LOC113072529	glutathione S-transferase theta-1-like isoform X2	up	2.08	0.0042
gene-LOC113055297	UDP-glucuronosyltransferase 1-5-like	up	3.63	1.23E-06
gene-LOC113112835	alcohol dehydrogenase class-3	up	3.35	5.34E-11
gene-LOC113120712	epoxide hydrolase 1-like	up	2.38	5.21E-05
**drug metabolism-cytochrome P450**				
gene-LOC113105784	flavin-containing monooxygenase FMO GS-OX5-like	up	3.47	4.67E-06
gene-LOC113121145	dimethylaniline monooxygenase [N-oxide-forming] 5-like	up	2.14	0.00033
gene-LOC113108170	amine oxidase [flavin-containing]-like isoform X1	up	2.93	3.75E-07
**drug metabolism-other enzymes**				
gene-LOC113038039	UMP-CMP kinase	down	-2.23	4.58E-05
gene-LOC113105628	cytidine deaminase	up	3.78	0.00029
gene-LOC113041190	cytidine deaminase-like	down	-3.70	0.00065
gene-LOC113115806	dihydropyrimidine dehydrogenase [NADP(+)]-like	up	2.35	6.26E-09
**other detoxification-related DEGs**				
gene-LOC113108503	cytochrome P450 27C1-like	up	2.64	0.00093
gene-LOC113120976	cytochrome P450 2J1-like	up	2.45	0.00014
gene-LOC113077208	cytochrome P450 2J6-like	down	-6.74	9.90E-15

gene-LOC113077392	cytochrome P450 2U1-like isoform X1	up	4.76	8.45E-06
gene-LOC113053331	cytochrome P450 27C1-like	up	2.70	7.06E-07
gene-LOC113051375	cytochrome P450 2U1	up	2.71	0.00573
gene-LOC113043250	cytochrome P450 2J6-like	up	3.21	8.47E-10
gene-LOC113044178	cytochrome P450 4V2-like isoform X1	up	2.56	2.07E-08
gene-LOC113062931	cytochrome P450 2U1-like isoform X1	up	4.97	1.84E-06
gene-LOC113108126	cytochrome P450 27C1-like	up	2.40	0.00229
Carassius_auratus_newGene_8844	cytochrome P450 3A24-like	up	2.40	9.35E-09
gene-LOC113051604	cytochrome P450 2C20-like	up	5.75	1.34E-09
gene-LOC113083757	cytochrome P450 3A24-like	up	2.20	0.00044
gene-LOC113049886	cytochrome P450 1A1-like	up	2.32	0.0003
gene-LOC113120974	cytochrome P450 2J6-like	up	2.13	9.20E-05
gene-LOC113115411	cytochrome P450 2C8	up	3.58	0.00014
gene-LOC113041251	cytochrome P450 2U1-like	down	-2.03	0.00045
gene-LOC113100639	cytochrome P450 3A24-like	up	2.24	7.13E-05
gene-LOC113043248	cytochrome P450 2J6-like	up	2.34	0.00586
gene-LOC113066727	cytochrome P450 2J6-like	up	3.10	5.34E-11
gene-LOC113038900	cytochrome P450 3A24-like	up	2.67	6.98E-08
